# STAPLED FASCIAL CLOSURE *VS*. CONTINUOUS HAND-SEWN SUTURE: EXPERIMENTAL STUDY OF THE ABDOMINAL WALL ON PORCINE MODEL AND HUMAN CADAVER

**DOI:** 10.1590/0102-672020240007e1800

**Published:** 2024-05-06

**Authors:** Francisco TUSTUMI, George Felipe Bezerra DARCE, Murillo Macedo LOBO, Ricardo Zugaib ABDALLA, Thiago Nogueira COSTA

**Affiliations:** 1 Universidade de São Paulo, Department of Gastroenterology - São Paulo (SP), Brazil

**Keywords:** Hernia, Abdominal Wall, Incisional Hernia, Ventral Hernia, Hérnia, Parede Abdominal, Hérnia Incisional, Hernia Ventral

## Abstract

**BACKGROUND:**

One of the primary complications associated with large incisions in abdominal surgery is the increased risk of fascial closure rupture and incisional hernia development. The choice of the fascial closure method and closing with minimal tension and trauma is crucial for optimal results, emphasizing the importance of uniform pressure along the suture line to withstand intra-abdominal pressure.

**AIMS:**

To evaluate the resistance to pressure and tension of stapled and sutured hand-sewn fascial closure in the abdominal wall.

**METHODS:**

Nine abdominal wall flaps from human cadavers and 12 pigs were used for the experimentation. An abdominal defect was induced after the resection of the abdominal wall and the creation of a flap in the cadaveric model and after performing a midline incision in the porcine models. The models were randomized into three groups. Group 1 was treated with a one-layer hand-sewn small bite suture, Group 2 was treated with a two-layer hand-sewn small bite suture, and Group 3 was treated with a two-layer stapled closure. Tension measurements were assessed in cadaveric models, and intra-abdominal pressure was measured in porcine models.

**RESULTS:**

In the human cadaveric model, the median threshold for fascial rupture was 300N (300-350) in Group 1, 400N (350-500) in Group 2, and 350N (300-380) in Group 3. Statistical comparisons revealed non-significant differences between Group 1 and Group 2 (p=0.072, p>0.05), Group 1 and Group 3 (p=0.346, p>0.05), and Group 2 and Group 3 (p=0.184, p>0.05). For porcine subjects, Group 1 showed a median pressure of 80 mmHg (85-105), Group 2 had a median of 92.5 mmHg (65-95), and Group 3 had a median of 102.5 mmHg (80-135). Statistical comparisons indicated non-significant differences between Group 1 and Group 2 (p=0.243, p>0.05), Group 1 and Group 3 (p=0.468, p>0.05), and Group 2 and Group 3 (p=0.083, p>0.05).

**CONCLUSIONS:**

Stapled and conventional suturing resist similar pressure and tension thresholds.

## RESUMO

**RACIONAL:**

Uma das principais complicações associadas a grandes incisões em cirurgia abdominal é o risco aumentado de ruptura do fechamento fascial e desenvolvimento de hérnia incisional. A escolha do método de fechamento fascial e o fechamento com tensão e trauma mínimos é crucial para resultados ideais, enfatizando a importância da pressão uniforme ao longo da linha de sutura, para suportar a pressão intra-abdominal.

**OBJETIVOS:**

Avaliar a resistência à pressão e tensão do fechamento fascial grampeado e suturado à mão na parede abdominal. **MÉTODOS:** Nove retalhos de parede abdominal de cadáveres humanos e 12 suínos foram empregados para experimentação. Um defeito abdominal foi induzido após a ressecção da parede abdominal e criação de um retalho no modelo cadavérico e após a realização de uma incisão na linha média nos modelos suínos. Os modelos foram randomizados em três grupos. O Grupo 1 foi tratado com uma sutura manual continua, em uma camada, o Grupo 2 foi tratado com uma sutura manual continua, em duas camadas e o Grupo 3 foi tratado com um fechamento grampeado de duas camadas. As medidas de tensão foram avaliadas em modelos cadavéricos e a pressão intra-abdominal foi medida em modelos suínos.

**RESULTADOS:**

No modelo cadavérico humano, o limiar médio para ruptura fascial foi de 300N (300-350) no Grupo 1, 400N (350-500) no Grupo 2 e 350N (300-380) no Grupo 3. As comparações estatísticas não revelaram diferenças significativas entre Grupo 1 e Grupo 2 (p=0,072, p>0,05), Grupo 1 e Grupo 3 (p=0,346, p>0,05) e Grupo 2 e Grupo 3 (p=0,184, p>0,05). Nos suínos, o Grupo 1 apresentou uma pressão mediana de 80 mmHg (85-105), o Grupo 2 teve uma mediana de 92,5 mmHg (65-95) e o Grupo 3 teve uma mediana de 102,5 mmHg (80-135). As comparações estatísticas indicaram diferenças não significativas entre Grupo 1 e Grupo 2 (p=0,243, p>0,05), Grupo 1 e Grupo 3 (p=0,468, p>0,05) e Grupo 2 e Grupo 3 (p=0,083, p>0,05).

**CONCLUSÕES:**

As suturas grampeadas e convencionais resistem a limiares de pressão e tensão semelhantes.

## INTRODUCTION

Despite the growing popularity of minimally invasive approaches in abdominal surgery, it is unlikely that the open approach will ever entirely disappear from the surgical landscape. Minimally invasive techniques, such as laparoscopy and robotic-assisted surgery, have indeed revolutionized many procedures, offering benefits such as less pain, shorter hospital stays, and faster recovery times^6^ ^,^ ^7^ ^,^ ^10^ ^,^ ^12^ ^,^ ^24^ ^,^ ^29^. However, the open approach continues to hold a crucial role, particularly in challenging cases that demand wide exposure.

One of the primary complications associated with large incisions in abdominal surgery is the increased risk of fascial closure rupture and hernia formation. A large incision inherently leads to greater tension during the closure of the fascial layers^18^ ^,^ ^21^. This heightened tension poses a substantial risk of fascial dehiscence, where the layers of the abdominal wall separate prematurely, favoring the development of incisional hernias. The incidence of hernias following major abdominal surgeries ranges from 2 to 20% across various studies. However, individuals with wound disorders may experience an incidence as high as 40%^26^. These complications can impact patients’ quality of life and usually demand additional surgical interventions, which implies higher treatment costs^25^.

Incisional hernia development depends on several risk factors, including non-modifiable factors like patient age and modifiable factors like technique and materials^11^. The choice of the fascial closure method and closing with minimal tension and trauma is crucial for optimal results, emphasizing the importance of uniform pressure along the closure line to withstand intra-abdominal pressure. Failure to adhere to these principles may lead to suture disruption, tissue tearing, and an elevated risk for postoperative hernias.

Employing an appropriate technique for suturing the aponeurosis post-laparotomy is crucial for preventing the formation of incisional hernias^9^. Numerous techniques are available, differing in suture types, point distances, and the number of closure layers^20^. While mass closure has historically been the prevailing method for median incision closure, experimental studies advocating the small bite technique have significantly reduced incisional hernia rates^3^. The Surgical Trial In Traumatic intraCerebral Haemorrhage (STITCH), a multicenter randomized controlled study, compared the small bites technique with mass closure, revealing a reduction in incisional hernia rates from 21 to 13% (p=0.022, p<0.05) in the small bites group^8^. The hypothesis is that the small bites technique reduces tissue trauma, achieving better tension distribution along the suture.

The choice of suture material is also critical, with slow-absorbing materials favored over fast-absorbing threads to minimize the risk of forming incisional hernias^22^. Some studies support the use of non-absorbable threads, indicating a lower incidence of incisional hernias with a minor risk of complications, suggesting a reduction of up to 32% compared to groups using absorbable materials, although a consensus has not yet been reached^13^.

Some authors have proposed using staplers to emulate small bites^1^ ^,^ ^19^. Theoretically, the principles of good-quality conventional closure methods can be replicated using surgical staplers. Stapling entails bringing together tissue edges using non-absorbable materials positioned at short intervals. This method distributes tension through successive staples, ensuring tissue apposition while minimizing bleeding and tissue damage^15^.

While the stapled technique has a theoretical basis, experimental studies comparing this method with the conventional abdominal wall closure technique are lacking. Thus, this study aimed to evaluate the resistance to pressure and tension of stapled and hand-sewn fascial closure in the abdominal wall.

## METHODS

Human cadaveric and porcine models were used for the experimentation and testing purposes. The research was conducted during October and November 2019 at the Experimental Surgery Research Center of the School of Medicine, University of São Paulo, and the Death Verification Service of the same institution. Ethical approval for the study was obtained from the local ethics committee (CAAE: 47765721.0.0000.0068).

### Study models

The study was conducted in fresh human cadavers and pigs.

Nine abdominal wall flaps from fresh-body human cadavers were utilized, devoid of ventral hernia and having no history of previous laparotomy. Only adults (18 to 65 years old) were included. Obese (body mass index >30 kg/m^2^) specimens were excluded.

Twelve male pigs (*Sus scrofa domesticus*) were evaluated. Animals weighed between 30 and 35 kg at four months of age. All specimens were initially anesthetized and euthanized.

### Surgical techniques

The procedures were performed in cadaveric and porcine models. Two experienced surgeons (T.N.C. and M.M.L.F.) conducted all the procedures.

**Figure 1 -:**
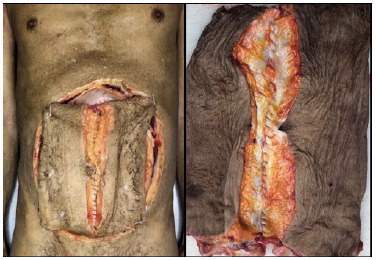
A resection of the anterior abdominal wall was performed in the cadaveric model, resulting in a comprehensive flap encompassing lateral abdominal wall muscles, spanning all layers from the epidermis to the peritoneum. The flap was resected with dimensions of 50 cm in the craniocaudal direction and at least 25 cm in the lateral-lateral direction. Following the flap resection, a median incision was made, exposing the aponeurosis through the section of the alba line.

In the cadaveric model, a resection of the anterior abdominal wall was performed, resulting in a comprehensive flap encompassing lateral abdominal wall muscles, spanning all layers from the epidermis to the peritoneum. The flap was resected with dimensions of 50 cm in the craniocaudal direction and at least 25 cm in the lateral-lateral direction. Following the flap resection, a median incision was made, exposing the aponeurosis through the section of the alba line (Figure 1).

In the porcine model, the animals were initially anesthetized (a combination of propofol and fentanyl) and euthanized with anesthesia overdose, and then a median incision was made.

**Figure 2 -:**
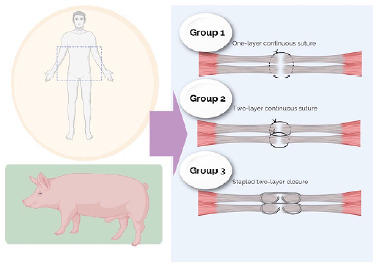
Abdominal wall flaps of fresh human cadavers and pigs were used for the experimentation. The models were randomized into three groups: Group 1 was treated with a one-layer continuous small-bite suture, Group 2 was treated with a two-layer continuous small-bite suture, and Group 3, with stapled fascial closure.

The animals and specimens were then randomized into three groups, with equal numbers of participants in each group (block randomization 1:1:1). After the section of the aponeurosis, a researcher not involved in the surgical interventions opened the envelope. The randomization was carried out separately for porcine and cadaveric models (Figure 2).

Group 1: The fascia was closed in a single layer with continuous hand-sewn suture using polydioxanone 0, employing the small bite technique.

Group 2: The fascia was closed in two layers (posterior and anterior sheaths of the rectus) with continuous hand-sewn suture, using polydioxanone 0, applying the small bite technique.

**Figure 3 -:**
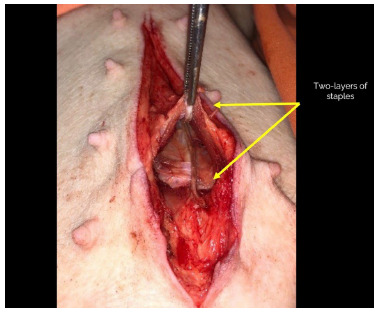
In Group 3, the fascia was closed in two layers with staples. A tunnel was performed on both sides of the alba line through the dissection of the space between the anterior sheath of the rectus and the fibers of the rectus muscle. After the tunnel was made, a stapler (GIA™ Stapler with Tri-Staple™ Technology, 60 mm Medium/Thick Stapler; Medtronic) was adjusted to apprehend the bilateral white line on the entire surface of the device, and then stapling was performed.

Group 3: The fascia was closed in two layers with staples (posterior and anterior sheaths of the rectus). A tunnel was performed on both sides of the alba line through the dissection of the space between the anterior sheath of the rectus and the fibers of the rectus muscle. After the tunnel was made, a stapler (GIA™ Stapler with Tri-Staple™ Technology, 60 mm Medium/Thick Stapler; Medtronic) was adjusted to apprehend the bilateral white line on the entire surface of the device, and then stapling was performed (Figure 3).

### Outcomes assessment

**Figure 4 -:**
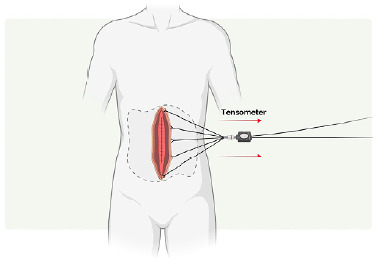
A tension measurement device was used after the closure of the aponeurosis for the cadaveric models. The abdominal wall flap was pulled on one side until there was evidence of rupture of the aponeurosis fibers while the tension in Newtons was measured (N).

For the cadaveric models, the abdominal wall flap was pulled on one side until the evidence of friction of the aponeurosis fibers, while the tension in Newtons was measured (N) by a tensometer, according to a previously published technique^30^ (Figure 4).

**Figure 5 -:**
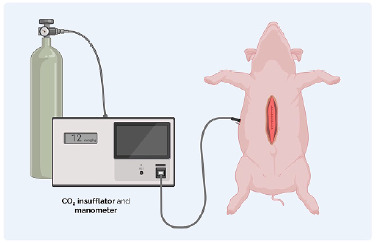
For the porcine models, after the closure of the aponeurosis, a 12 mm trocar was inserted in the animal’s left flank and a system of latex tubes with one way for insufflation of compressed air and another way connected to a tensometer. Carbon dioxide was inflated into the peritoneal cavity until there was evidence of friction of the aponeurosis fibers, while the pressure in the wall was measured in mmHg.

For the porcine models, after the closure of the aponeurosis, a 12 mm trocar was inserted into the animal’s left flank, and an insufflator system was connected (Insufflator Stryker Pneumosure 45L). Carbon dioxide was inflated into the peritoneal cavity until there was evidence of friction of the aponeurosis fibers, while the pressure in the wall was measured in mmHg by an internal system manometer (Figure 5).

### Statistical analysis

Comparisons between independent groups were performed using the Mann-Whitney non-parametric test with a one-tailed alternative hypothesis. The level of significance adopted was 5% for all hypothesis tests. The analyses were performed using Statistical Package for Social Sciences (SPSS) v. 25 for Windows.

## RESULTS

The characteristics of the specimens and animals are described in Table 1.

**Table 1 -:** Characteristics of the specimens and animals.

Human cadaveric model					
Specimen	Age	Weight (kg)	Gender	Type of fascial closure	Tension (N)
1	62	74	Male	One layer	300
2	59	64	Male	One layer	300
3	64	65	Male	One layer	350
4	63	60	Female	Two layers	500
5	58	60	Female	Two layers	350
6	63	56	Female	Two layers	400
7	57	44	Male	Stapled	300
8	64	62	Female	Stapled	350
9	61	70	Female	Stapled	380
**Porcine model**					
**Animal**			**Gender**	**Type of fascial closure**	**Pressure (mmHg)**
1			Male	One layer	85
2			Male	One layer	95
3			Male	One layer	105
4			Male	One layer	90
5			Male	Two layers	90
6			Male	Two layers	95
7			Male	Two layers	70
8			Male	Two layers	65
9			Male	Stapled	100
10			Male	Stapled	105
11			Male	Stapled	80
12			Male	Stapled	135

Table 2 presents data from the three groups (Group 1, Group 2, and Group 3) based on different parameters in human cadaveric and porcine subjects. In the human cadaveric models, Group 1 had a median value of 300 (range: 300-350), Group 2 had a median value of 400 (range: 350-500), and Group 3 had a median value of 350 (range: 300-380). Statistical comparisons revealed non-significant differences between Group 1 and Group 2 (p=0.072, p>0.05), Group 1 and Group 3 (p=0.346, p>0.05), and Group 2 and Group 3 (p=0.184, p>0.05).

**Table 2 -:** Median tension (N) and pressure (mmHg), with the corresponding range (minimum and maximum), for the human cadaveric and porcine models. The p-values for the comparisons (Mann-Whitney U test) were presented.

	Groups			Comparisons (p-value)		
	Group 1 (one layer)	Group 2 (two layers)	Group 3 (stapled)	Group 1 vs. 2	Group 1 vs. 3	Group 2 vs. 3
Human cadaveric model (N)	300 (300-350)	400 (350-500)	350 (300-380)	0.072	0.346	0.184
Porcine (mmHg)	80 (85-105)	92.5 (65-95)	102.5 (80-135)	0.243	0.468	0.083

For porcine subjects, Group 1 showed a median pressure of 80.0 mmHg (range: 85-105), Group 2 had a median of 92.5 mmHg (range: 65-95), and Group 3 had a median of 102.5 mmHg (range: 80-135). Statistical comparisons indicated non-significant differences between Group 1 and Group 2 (p=0.243, p>0.05), Group 1 and Group 3 (p=0.468, p>0.05), and Group 2 and Group 3 (p=0.083, p>0.05).

## DISCUSSION

This experimental study, using cadavers and pigs, successfully demonstrated the viability and reproducibility of these models for aponeurosis closure techniques using hand-sewn sutures and a stapler. The findings of this study indicated similar tension and pressure thresholds among conventional techniques and stapled fascial closure.

Using staplers for aponeurosis in surgical procedures offers several notable advantages. Firstly, staplers can potentially reduce operative time, enhancing overall procedural efficiency significantly. Honório et al.^14^, in a meta-analysis comparing hand-sewn and stapled anastomosis for gastrectomy, found that staplers save 22 minutes of operation time on average. Similar findings can be seen in other studies comparing stapled and hand-sewn anastomosis for other types of surgery^16^ ^,^ ^23^. This time-saving aspect is crucial in minimizing patient exposure to anesthesia and decreasing the risk of complications associated with prolonged surgical interventions and prolonged abdominal viscera exposure, which might be even more relevant in critical patients or urgent surgeries. Shorter surgical interventions alleviate overall physiological stress on patients, fostering quicker recovery and diminishing postoperative complications, including infection. In a meta-analysis conducted by Cheng et al.^5^, it was demonstrated that there is a 14% increase in the probability of complications for each additional 30 minutes of operating time. The probability of surgical site infection rises with time intervals, with a corresponding increased likelihood of 13, 17, and 37% for every 15 minutes, 30 minutes, and 60 minutes of surgery, respectively^4^. Operating room time exceeding 100 minutes in urgent surgeries is linked to a higher risk of developing deep vein thrombosis and pulmonary embolism, exhibiting a 7 and 5% respective increase for every additional 10 minutes beyond the initial 100 minutes^27^. In addition, the use of staplers aligns with the evolving paradigm of efficient procedures with decreased manipulation of tissues. Unnecessary manipulation of intraperitoneal organs in the abdomen contributes to increased adhesion formation^28^.

Reducing operative time contributes to improved patient outcomes and potentially holds economic benefits by lowering resource consumption, reducing postoperative complications, and elevating operative efficiency^5^. The efficiency gained through shorter surgeries can accommodate a higher volume of procedures, enhancing overall healthcare system productivity and reducing patient wait times for interventions.

Moreover, using staplers may reduce variability in surgical outcomes related to individual surgeons’ dexterity, as the standardized application of staples can lead to more consistent results across different practitioners. Kim et al.^17^, comparing hand-sewn and stapled gastric anastomosis, found that the hand-sewn group had higher variability in operation time than stapled anastomosis. Dissimilarities in surgical hand-sewn techniques can be linked to variations in surgical expertise and produce heterogeneous outcomes^32^.

Our study used fascial rupture and resistance to pressure and tension as the main outcomes for defining the efficacy of a suture line. While other variables like tissue perfusion and contamination contribute to fascial dehiscence, tension and pressure on sutures are major driving variables^18^. In this context, interventions to promote reduced abdominal pressure, including botulinum toxin and component separation, have a significant role in achieving tension-free midline fascial approximation and reducing the risk of hernia development.

Cakmak et al.^2^, in an experimental study with rats, investigated the effects of local botulinum A toxin injection on abdominal wall muscles. The authors found that, after three days, intra-abdominal capacitance was higher in rats that received botulinum toxin injection. Additionally, these rats exhibited significantly lower mean motor unit potential amplitude and duration in the rectus muscles. These findings suggest that local botulinum A toxin injection induces paralysis in abdominal wall muscles, leading to increased intra-abdominal volume and decreased pressure.

Wegdam et al.^33^ conducted a systematic review of prehabilitation with botulinum toxin injection under ultrasound guidance. The authors found that botulinum toxin has the potential to promote lateral abdominal wall muscle elongation, easy fascial closure, and avoid hernia formation.

While staplers offer notable advantages, it is essential to consider potential downsides associated with their use in aponeurosis closure. One significant drawback is the higher cost compared to traditional suture lines. Staplers can be more expensive, leading to an increase in the overall cost of surgery^31^. This financial aspect may be crucial, especially in healthcare settings where funding plays a pivotal role in decision-making. Only future high-quality studies will provide evidence for the best cost-effective option.

However, it is imperative to acknowledge the limitations of this study, particularly concerning long-term follow-up. The investigation does not address the long-term durability of stapled aponeurosis closures or potential complications, such as granuloma formation or chronic pain. In addition, the small sample size or inherent variability in the experimental models might contribute to the non-significant p-values and should be also acknowledged as a limitation. Furthermore, exploring potential factors influencing pressure values, such as anatomical variations, could provide additional context to interpret the results comprehensively. Future studies may benefit from larger sample sizes or alternative methodologies to validate and expand the findings of this study.

## CONCLUSIONS

Stapled and conventional suturing resist similar pressure and tension thresholds. The comparable outcomes achieved with the stapler suggest that this technique may emerge as a viable option for aponeurosis closure following laparotomy as well as a potential for addressing ventral hernias.
